# Operative vs. Mechanical Dentistry

**Published:** 1880-07

**Authors:** J. Allen Osmun

**Affiliations:** Newark, New Jersey


ARTICLE IV.

### OPERATIVE vs. MECHANICAL DENTISTRY.

BY DR. J. ALLEN OSMUN, NEWARK, NEW JERSEY.

We will in the first place consider the aim of operative dentistry—its successes, its failures, The aim of operative dentistry is the complete restoration of diseased and cavited teeth to a normal condition, so far as can be accomplished by art. That the aim is high and noble admits of no argument, and is worthy of all honor. And the profession is to be congratulated on the success that has been achieved. But the time comes when the best efforts often result in failure. Imperfect organizations, acquired or inherited disease, accidents, improper dental operations, neglect on the part of the patient, absorption of alveolus, and many kindred diseases tend to produce failure in operative dentistry. If this be true, than mechanical dentistry must be of importance and has a sphere to fill in the alleviation of the misfortunes of the human race, and yet of all the articles that are written and published, the bulk of them treat of operative dentistry, of cohesive vs. soft foil, permanent separation vs. Contour, the extraction of the sixth year molars, and many other articles of much interest and profit to the profession. But while the patient undergoing all of these different operations for the preservation of the natural organs, many of them have to resort to artificial substitutes as the result of the *very best practice.* The question naturally presents itself, why does not mechanical dentistry incite more interest, is it because that it does not require any skill to insert a Denture? Or is it that there is no demand for skillful treatment and care in this direction? It is well to take a retrospect of this branch of dental acquirements, and see if it is not possible to have a "new departure" in this direction. Mechanical dentistry is in a deplorable condition, receiving little or no encouragement from the "lights" of our profession, and to use their favorite expression they have "washed their hands of it" or that they "make no sets of teeth." There is a demand for mechanical dentistry of the highest order, and we must supply this demand, or fail in our efforts to be what we profess *we are;* many persons are thrown entirely on this branch, for the means of sustaining life and comfort. I know many will say, that they ought not to have neglected their teeth in the beginning and then they would not have had to resort to artificial substitutes; this sounds well, it is sentimental and pleasant to the ear, but people will be careless to the end of the world, it has always been so and is likely to remain so, and this fact standing out bold and clear, leaves no other alternative but to meet it with the best face we can, and devise means to make as complete a restoration as possible. Operative dentistry in its restricted sense, is but mechanical dentistry in a narrow sphere. Let us look at a few of the items that must be taken into consideration in the successful accomplishment of introducing a dention that will meet all the requirements of nature. A knowledge of physiology should be had in this branch as much as in the other branch of operative dentistry, so as to properly consider the place it occupies in the economy with its frame work of bones, walls of hard and soft tissues, organs of speech, &c., that when any of their teeth are lost, to replace them skillfully so the lost functions are fully restored; in order to determine what base shall be used, without interfering with the physiological parts involved, for often we come across sad evidences of the neglect of this precaution. Physiognomy should receive attention, so that the features are not distorted and the "human face divine" made hideous; for often we meet with cases where we can see so many contradictory evidences of character, the direct result of improperly made dentions; where all the requirements of this knowledge is prevented, then the laws that govern speech also should be carefully considered, that clear connection may result; we have to consider how long the teeth should be, the inclination, separation one from another, how far back they can extend on the palate, how full to restore the natural expression, size and color to preserve the harmony of temperaments, and many other points that must be carefully looked after in order to meet all the manifold deviations that come under our care. Does not the mere filling of a tooth sink into insignificance when compared to true dental prosthetis. I venture to assert that it requires more skill to properly construct and insert a dention than to put in the most difficult gold filling, that a wider range of knowledge is required, ought to be exercised, that as much comfort and happiness is to be obtained from the successful accomplishment of this speculation as any which comes within the provision of the dentist. I can well appreciate the feelings of the thoughtful and intelligent operator, when every dental substitute he introduces is a source of mortification and sorrow, that the science he is practicing has failed to reach his "*ultima thule*" in preserving the organs of mastication given to man by a wise Creator, no matter how they have come to this sad end, either by neglect of patient or unskillful treatment; hence he has furnished the "*sample sets*" in the little glass case, and the true operator who has the good of his patients at heart, does not urge his patrons "to take gas" have the old teeth out and get a new set, but uses all the skill and care he can to preserve that which nature has given to man, but when they are gone, he also, if he be true to his calling, bestows the *same care* and skill in restoring the lost functions, as he would have in preserving them. What is more noble than to restore the expression to a face which has lost every lineament of former beauty and intelligence, this is the province of the mechanical dentist; hence the separation of the branches of our specialty should not be encouraged, for the mere artisan, no matter how skillful he may be, is not competent to take the place of the surgeon dentist whose every operation tends to make him more successful in copying nature's handiwork.

